# Comparison of Accelerometer-Based Cut-Points for Children’s Physical Activity: Counts vs. Steps

**DOI:** 10.3390/children5080105

**Published:** 2018-08-03

**Authors:** Cheryl A. Howe, Kimberly A. Clevenger, Ryann E. Leslie, Moira A. Ragan

**Affiliations:** 1 School of Applied Health Sciences and Wellness, Ohio University, 1 University Terrace, Grover Center E154, Athens, OH 45701, USA; leslie25@live.marshall.edu; 2 Department of Kinesiology, Michigan State University, East Lansing, MI 48824, USA; cleven18@msu.edu; 3 Gladys W. and David H. Patton College of Education, Ohio University, Athens, OH 45701, USA; mr143312@ohio.edu

**Keywords:** pediatric, accelerometry, cadence, step rate, energy expenditure, free-play

## Abstract

*Background:* Accelerometers measure complex movements of children’s free play moderate-vigorous physical activity (MVPA), including step and non-step movements. Current accelerometer technology has introduced algorithms to measure steps, along with counts. Precise interpretation of accelerometer-based cadence (steps/min) cut-points is necessary for accurately measuring and tracking children’s MVPA. The purpose of this study was to assess the relationships and agreement between accelerometer-based cut-points (cadence and counts/min) to estimate children’s MVPA compared to measured values. *Methods*: Forty children (8–12 years; 25 boys) played 6–10 games while wearing a portable metabolic analyzer and GT3X^+^ to measure and estimate MVPA, respectively. Correlation, kappa, sensitivity, and specificity assessed the relationships and agreement between measured and estimated MVPA. *Results*: Games elicited, on average, 6.3 ± 1.6 METs, 64.5 ± 24.7 steps/min, and 3318 ± 1262 vertical (V) and 5350 ± 1547 vector-magnitude (VM) counts/min. The relationship between measured and estimated MVPA intensity was higher for cadence (*r* = 0.50) than V and VM counts/min (*r* = 0.38 for both). Agreement using V and VM counts/min for measuring PA intensity varied by cut-points (range: 6.8% (κ = −0.02) to 97.6% (κ = 0.49)), while agreement was low using cadence cut-points (range: 4.0% (κ = 0.0009) to 11.3% (κ = 0.001)). *Conclusion*: While measured and estimated values were well correlated, using cadence tended to misclassify children’s free-play MVPA.

## 1. Introduction

Accurately measuring physical activity (PA) is necessary to determine if children acquire sufficient moderate-to-vigorous PA (MVPA) to promote healthy growth patterns and lifestyles. Obtaining true measures of MVPA in children is challenging due to the unpredictable and intermittent nature of play behavior [1]. Objective monitors, such as accelerometers and pedometers, have continued to advance in terms of technology and interpretation, especially over the past decade. Currently, numerous accelerometer-based (counts/min) prediction equations and cut-points are available for estimating children’s PA energy expenditure (PAEE) and PA intensity [2,3,4]. However, the inconsistency in the literature (i.e., correlations between cut-points and measured PAEE and PA intensity range from *r* = 0.36 to *r* = 0.92), is attributed to both the nature of children’s play and the use of constrained (i.e., treadmill walking/running) rather than unconstrained play [5,6]. While researchers continue to explore valid methods for interpreting accelerometer signals to accurately measure PA, the use of research-grade activity monitors is not practical for measuring and tracking daily PA by non-researchers.

Alternatively, pedometers are activity monitors that simply measure steps, a unit of PA measure that is easier to interpret by the general population. Pedometers have shifted from spring-lever mechanisms to uniaxial or triaxial accelerometry, technology that improves the sensitivity to movement at slower paces and provides a time-stamp to produce data in shorter epoch lengths (i.e., steps/min, instead of steps/days). Combining this simple PA measure with the ease of use, low cost, PA-promoting capacity of the digital feedback, and improved technology, it is no wonder pedometers continue to rise in popularity and everyday use by the general population and practitioners. Additionally, there is a great deal of evidence on the reliability and validity for the use of pedometers in PA research [7,8].

Based on the current research, step recommendations for children range from 11,000–12,000 steps/day for girls and 13,000–15,000 steps/day for boys [9,10,11,12]. While these recommendations provide children with daily step goals, it has been reported that children who acquire these goals have an increased likelihood of meeting the daily 60-min MVPA recommendation [13]. One of the first cadence-calibration studies by Harrington et al. (2012) argued against the use of steps/day to estimate time spent in MVPA, stating it did not confirm actual bouts of MVPA throughout the day [14]. Although new pedometers have potential, there is little research on the development of steps/min (i.e., cadence) cut-points for measuring children’s daily MVPA [14,15,16,17,18,19]. Providing a means of classifying children’s cadence, rather than total daily steps, may allow practitioners, physical education teachers, and even parents to more accurately assess children’s daily MVPA.

From the limited available research, cadence cut-points for children range from 102 to 140 steps/min for MVPA [14,15,16,17,18,19]. For example, Saunders et al. (2014) used an accelerometer-based pedometer (Walk4Life MVP; Walk4Life, Inc, Oswego, IL, USA) and a cut-point of ≥100 steps/min for estimating time spent in MVPA during treadmill walking [16]. Using this same cut-point, there was a strong relationship between estimated and measured PA intensity (R^2^ = 0.69) with high sensitivity (97.7%) and specificity (87.7%). Another study by Harrington et al. (2012) used an accelerometer-based activity monitor (ActivPAL; PAL Technologies, Glasgow, Scotland, UK) to measure children’s repetitive or rhythmic movements patterns (e.g., treadmill or over-ground walking and jogging) [14]. This study also used a cut-point of ≥100 steps/min for MVPA, which was used in the interpretation of the 2005–2006 National Health and Nutrition Examination Study (NHANES) PA data to reveal that youth (*n* = 2610) spent only ~12 min/day in MVPA [20]. More recently, using a constrained treadmill protocol of varying speeds in 6–20 year old youths, the threshold for MVPA derived from direct observation, rather than from an activity monitor, ranged from 128 steps/min in the youngest youth (6-8 years) to 101 steps/min in older youth (15–17 years) [19].

From these studies, a range of 100–128 steps/min is considered to be valid for estimating children’s constrained MVPA. Research needs to expand on these studies to determine accelerometer-based cadence cut-points for interpreting children’s unconstrained play behavior [14,16]. Children’s play is complex and difficult to interpret, especially when using cut-points that were developed from and tested on repetitive-stepping movement patterns observed during constrained (treadmill) walking or running. Therefore, the primary purpose of this study was to assess the accuracy of established accelerometer-based cadence cut-points for correctly classifying children’s free-play MVPA. The secondary purpose was to compare the relationship between measured and estimated PA intensity using cadence and vertical (V) and vector-magnitude (VM) counts/min cut-points of children’s play behavior.

## 2. Materials and Methods

### 2.1. Recruitment

Third- through fifth-grade children (8–13 years) from five elementary schools in Athens County, Ohio, were recruited for this study over a 2-year period from 2011–2013. Upon arrival at the lab, height and weight were measured to determine body mass index (BMI). Recruitment was stratified by sex and BMI in attempts to enroll equal samples of boys and girls and healthy weight (HW = BMI <85th percentile) and overweight or obese (OW = BMI ≥85th percentile) children. Eligible children were free from cardiorespiratory, metabolic, neurological, or physical impairments and were not taking any medications that would either prevent them from being physically active or affect metabolism (e.g., Concerta, Ritalin, etc.). Each eligible child and related parent/guardian signed the approved informed assent/consent documents in accordance with the Declaration of Helsinki and Ohio University’s Institutional Review Board regulations (IRB# 11F018).

### 2.2. Testing Protocol

A detailed description of the testing protocol has been previously published [21]. Briefly, a menu of 30 games was selected by a focus group of local physical education teachers based on their overall activity level, age-appropriateness, enjoyment, and feasibility for inclusion in the study. The children reported to the lab following a 3-h fast at which time their stature (m) and weight (kg) were measured to determine weight status using age- and sex-specific BMI growth charts [22]. Resting metabolic rate (RMR) was measured with the MedGem^®^ analyzer (MicroLife USA, Clearwater, FL, USA) to determine baseline energy expenditure (EE) following standard procedures [23]. Following RMR measurements, each child ate a small snack (~150 kcal) prior to playing up to 10 children’s games for six minutes each in a large gymnasium. From the menu of 30 proposed moderate-vigorous intensity games, an online random number-generator was used to select 10 games in random order for each child. The testing protocol, including resting and exercise measures, lasted approximately 2.5 to 3.0 h.

During the study visit, each child was accompanied by a sibling or friend of similar age for social support and to simulate a more natural play environment during the games. To ensure a sufficient number of players for each game or activity, undergraduate students, who were extensively trained to match children’s PA intensity and skill-level, joined the children in playing games that required more than two players (i.e., tag, sharks and minnows, soccer, etc.). Games were played in random order for 6 min, with 4 min rest between games. An elongated rest period (15–30 min) was provided after game five to ensure the child had adequate rest and to recalibrate the metabolic cart. Data from the first minute of each game, during which the children were becoming comfortable playing the game, were not included in the analyses. The final 10 s of each game were also omitted due to obvious changes in behavior during the countdown to stopping each game. After these omissions, 4:50 of data for each game were used in the analyses. Similar protocols have been used previously in measuring children’s play. Due to the intermittent nature of children’s play, during which children will not necessarily reach steady state, average PAEE and intensity was calculated rather than “steady state” values for each game [24,25]. Children were encouraged to play as they would naturally play in the school gymnasium or playground, allowing them to stop and start throughout the game at will.

The children played an average of 9.5 games (range: 6–10 games). One child volunteered for only 6 games, while 11 other children participated in fewer than 10 games because of time constraints or equipment failures. Only games with all 6 min of data were included in the analyses. This resulted in a total of 380 game sessions played with each game played an average of 13 times (range: 11 to 17 times) by different children.

### 2.3. Criterion Measure

During the games, children wore a portable metabolic analyzer (Oxycon™ Mobile, Vyaire Medical, Mettawa, IL, USA) for breath-by-breath gas analysis [26,27]. A POLAR heart rate monitor strap was secured to the child’s chest to monitor heart rate throughout the activities. PAEE, the amount of PA-related EE above RMR, was calculated as total EE minus individually measured RMR. Average PAEE (kcal/min) was computed for each game using the previously described 4:50 of breath-by-breath data. Measured PA intensity of the games, expressed as a multiple of individually-measured RMR (Youth-MET), was classified using three METs as the cut-point for MVPA [28].

### 2.4. Physical Activity Measurements

**Figure 1:**
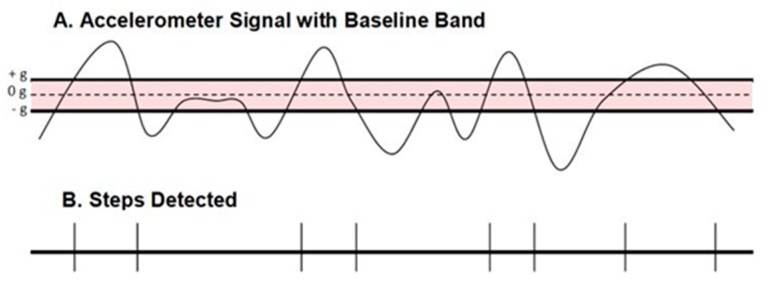
Crossing Threshold Mode using the accelerometer raw signal (**A**) for counting steps; (**B**) A step is detected when the signal passes beyond the baseline band (**–––**) and crossed the midline (----) of the band (ActiGraph, LLC.).

The GT3X^+^ (ActiGraph, Pensacola, FL, USA) worn on the right hip was used to assess PA intensity in steps/min. GT3X^+^ has been validated for use in children for both V and VM counts/min [4,29,30,31]. Using ActiLife software (version 6.5.3; ActiGraph, Pensacola, FL, USA), GT3X^+^ monitors were initialized to record activity at a sampling rate of 30 Hz and downloaded using a 1 s epoch. A second POLAR heart rate watch was synchronized with the same laptop computer as the GT3X^+^ and was used to time the activities to allow for time-synchronization of Oxycon and ActiGraph data. Using the crossing threshold mode, a step was registered by the GT3X^+^ as the sigmoidal curve of the accelerometer signal that crosses through the midline (0 gravity; g) and then outside of either the +g or −g baseline reference bands (Figure 1; ActiGraph, LLC^©^). This method of determining steps using the GT3X^+^ has not been validated for estimating children’s PAEE or PA intensity.

GT3X^+^ accelerometer data were recorded for both V and VM in counts/sec. Average V and VM counts/sec and cadence (steps/min) were calculated for each game using the same time period as the metabolic data (described above) and established cut-points were used for classifying PA intensity [4,29,30,31]. Freedson’s (age- and sex-specific) and Evenson’s counts/min (converted from counts/15 s) cut-points were recommended for this population to classify PA intensity from V counts/min [3,32]. The available cut-points for classifying PA intensity from VM counts/min in this population were published by Zhu et al. (2013) and Hanggi et al. (2013) [30,31]. Available cadence cut-points (range: 100–109 steps/min) for classifying children’s MVPA from pedometer studies were published by Saunders et al. (2014) and Harrington et al. (2012) [14,16]. Based on the consensus of this previous research and the lack of validated cadence cut-points, conservative MVPA cut-points of 100 and 109 steps/min were selected to assess accuracy in classifying MVPA. The accelerometer counts/min and cadence cut-points used are listed in Table 1.

### 2.5. Statistics

A mixed-model ANOVA was used to assess differences in PAEE (kcal/min), PA intensity (METs), V and VM counts/min, and steps/min with sex, weight status, and game as main effects. Child, embedded within game, was included in the model as a random factor to account for inter- and intra-individual variability, avoiding inflation of Type 1 error in analyzing 380 outcomes from a smaller sample size. The accuracy of estimated PA intensity classification (MVPA or not) from V and VM counts/min and steps/min, compared to the measured PA intensity classification was tested using Cohen’s Kappa coefficients, as well as the sensitivity (Se%) and specificity (Sp%). The Pearson correlation coefficient (*r*) was used to assess the relationship between PAEE (kcal/min) and PA intensity (METs) and V and VM counts/min and steps/min. A significance level of *p* < 0.05 was used in all analyses, adjusted for multiple comparisons using the Bonferroni correction (Statistical package: SAS, version 9.4).

## 3. Results

### 3.1. Participants

Forty children volunteered to participate in the study (25 boys; 29 HW children). Girls were significantly heavier than boys, while OW children were heavier and had greater BMI percentile and higher RMR compared to HW children, as expected. (Table 2).

### 3.2. Measured PAEE and PA Intensity

Measures of PAEE are reported in Table 3 for all games and children combined (40 children × 9.5 games = 380 individual games sessions), as well as by sex and weight status. Individual game data can be found in supplemental materials (See Table A1, Appendix A. SDC 1, Physical Activity Energy Expenditure, Intensity, and Counts and Cadence by Game).

As presented in Table 3, when averaging the PA intensity of all of the games combined, collectively they were classified as MVPA (6.3 ± 0.1 METs), ranging from 4.4 ± 0.2 METs for *Hoop Stations*, which consisted of the children moving through stations to accomplish different tasks with one or more hula hoops, to 7.7 ± 0.7 METs for *Me and My Shadow*, a soccer follow-the-leader game using soccer dribbling skills (Appendix A). There were no differences in average METs across sex or weight status (*p* > 0.05).

### 3.3. Accelerometer-Based Counts and Steps

The average V counts/min for all games was 3335 ± 67 counts/min, ranging from 2092 ± 223 counts/min for *Sharks and Minnows*, a game of back and forth tag within a confined space, to 5135 ± 323 counts/min for *Slap Ball*, a modified game of hand ball. The average VM counts/min across games was 5350 ± 79 counts/min, ranging from 3944 ± 344 counts/min for *Sharks and Minnows* to 8037 ± 379 counts/min for *Clean Your Room*, where children keep their side of the court clean by tossing items to their opponent’s side. The average cadence across all games was 66.5 ± 1.3 steps/min, ranging from 32.9 ± 4.1 steps/min for *Hoop Stations* to 102.0 ± 4.0 steps/min for *Joker’s Wild*, a relay game of chance. Boys and HW children elicited 8.6–16.7% higher values across all three accelerometer-based PA measures compared to girls and OW children (*p* < 0.05).

### 3.4. Measured vs. Estimated PA Intensity

Correlation (*r*), inter-rater agreement (κ), Se%, and Sp% data are presented in Table 4 and Table 5. There was a stronger relationship, inter-rater agreement, and Se% for the Freedson cut-points compared to the Evenson cut-point in classifying children’s PA intensity. When using published VM cut-points by Hanggi et al. (2013) and Zhu et al. (2013), Zhu’s cut-point demonstrated a stronger relationship, inter-rater agreement, and Se% for estimating PA intensity compared to Hanggi’s cut-points [31,33]. Although a modest relationship exists among steps and measured PA intensity (*r* = 0.50) when using 100 steps/min cut-point, only 1 (i.e., *Joker’s Wild*) of the 30 games met this criterion for being classified as MVPA. In examining the metabolic data for each of the individual 380 game sessions played by the children individually, only 3 game sessions were classified as light-intensity PA with the remaining 377 games classified as MVPA.

## 4. Discussion

This study assessed the accuracy of established accelerometer-based cadence cut-points for estimating children’s PAEE and PA intensity and compared these cadence cut-points to established accelerometer-based counts/min cut-points for accurately classifying the intensity of children’s MVPA. Based on the age-specific Freedson V counts/min cut-points [2], 371 (97.6%) of the 377 games were correctly classified as MVPA, whereas based on the Evenson cut-point [4], only 298 (78.4%) of the games were correctly classified as MVPA with 78 (20.5%) of the games misclassified as light-intensity PA. However, Zhu’s and Hanggi’s VM counts/min cut-points resulted in 367 (96.6%) and 26 (6.8%) of the games being classified as MVPA, respectfully [30,31]. Finally, Harrington’s and Saunders’ cadence cut-points resulted in only 43 (11.3%) and 15 (4.0%) of the games classified as MVPA, respectively [14,16].

Using common unconstrained play activities proposed to be moderate-vigorous intensity, a modest relationship (*r* = 0.50) was found among cadence (≥100 steps/min) and measured PAEE and PA intensity. However, using the ≥100 steps/min MVPA cut-point, albeit the lowest available cut-point in the literature (range: 100–140 steps/min) [14,16,19], resulted in all but 1 of the games being misclassified as light intensity PA, instead of MVPA. If we had incorporated higher cadence cut-points, as available in the literature (e.g., 140 steps/min), this would have increased the error in classifying MVPA intensity.

In examining the accuracy of published V and VM counts/min cut-points for classifying children’s PA intensity, the Freedson [29] and Zhu [30] cut-points were superior over cut-points developed by Evenson [4] and Hanggi [31]. The discrepancies in accuracy of activity monitors is often blamed on a mismatch between the activities used to develop the cut-points and the activities the cut-points are being used to measure. However, Alhassan et al. (2012) found the Freedson age- and sex-specific cut-points, which were developed using primarily constrained treadmill activities, accurately estimated the PAEE of five self-paced activities (walking, bicycling, basketball, crafts, and Wii tennis) of varying intensities in 8–12-year-old children using the ActiGraph GT1M [34]. Conversely, the Evenson cut-points, which were developed using a wide range of activities types and intensities (coloring, basketball, jumping jacks, running, bicycling, etc.), did poorly in classifying children’s PA intensity in the current study, which was primarily MVPA. Yet Trost et al. (2011) found that both Evenson (κ = 0.68, Se% = 89.9%, Sp% = 89.2%, receiver operating characteristics (ROC) = 0.90), and Freedson (κ = 0.66, Se% = 88.3%, Sp% = 91.7%, ROC = 0.90) cut-points demonstrated significantly better agreement with measured PA intensity and better accuracy in classifying MVPA compared to other established cut-points (i.e., Puyau, Trueth, and Mattocks) across 12 standardized sedentary, lifestyle, and ambulatory activities [3]. The research is clear that counts/min cut-point accuracy and agreement varies across studies, indicating that no single set of cut-points will accurately measure all PA types and intensities, especially in children. That being said, care should be taken to use cut-points that were developed using similar activities as those being measured because, as observed in the current study, using accelerometer cut-points that were developed on activities with a greater range in PA intensity increases the opportunity for misclassifications when tested on MVPA alone. Furthermore, the comparison of previous research is limited because studies cited herein have also used a variety of methodologies, including different activity monitors and epochs (i.e., time lengths measured) to study activity. Varying devices also leads to different algorithms for converting the acceleration signal into counts and steps, while varying epoch lengths can alter the capacity to detect short bursts of high intensity PA [35].

While many prediction equations and cut-points exist to estimate and classify children’s PAEE and PA intensity from accelerometer-based counts/min, none of them do well in a free-play setting [36,37]. Even fewer cadence cut-points exist in the literature, especially those developed on accelerometer-based activity monitors and there is little evidence of their accuracy [14,16,18]. This study demonstrates that new accelerometry-based pedometers are moderately correlated with measured play intensity, indicating that they may have the potential to accurately measure children’s play behavior, although further study is required to support this conclusion. Specifically, more research needs to incorporate children’s unconstrained play behavior in future validation studies to identify a more accurate cadence cut-point to correctly classify MVPA during play. This study used activities proposed to be MVPA, which limits the PA intensity range to develop new cut-points for these devices. Replicating the current study to include larger sample sizes of children of all ages completing a wide variety of unconstrained play activities is needed to produce these cut-points.

Another limitation of the current study and the potential for developing a single set of cut-points to accurately measure children’s play behavior is the variability in the methods used to determining a step from the accelerometer signals. The ActiGraph GT3X^+^ used in the current study uses the “crossing threshold mode” to determine a step. The raw acceleration signal must cross this seemingly arbitrary value on either side of zero g to register as a step, using the same threshold across all populations. In referencing the work of Freedson’s lab, they created a prediction equation that uses different count values, a representation of the magnitude of the raw acceleration signal, for children relative to their age [29]. This means that younger children generate lower acceleration signal patterns than older children and adults. Future studies are needed to determine if adjusting this threshold by age, or perhaps other factors such as sex, weight and height, might improve the accuracy in detecting steps during children’s play.

## 5. Conclusions

When using accelerometers to measure or track children’s free play MVPA, accelerometer-based cadence cut-points have a stronger correlation with measured PA intensity, but are less accurate in correctly classifying MVPA, compared to counts/min. Therefore, more research is needed to either define appropriate cut-points or improve on how accelerometers and pedometers determine steps for measuring children’s play. With new pedometers using accelerometer-based technology, these devices have the potential to provide accurate PA information while maintaining the many features that attract their everyday use in the general population.

## Figures and Tables

**Table 1:** Cut-points for Classifying Children’s moderate-vigorous physical activity (MVPA).

Reference	Sample (Age)	MVPA Cut-Points	Derived From
		**counts/min**	
Freedson et al. (1997) *	80 (6–17 years)	≥803–2068 counts/min	Vertical axis
			(ActiGraph 7164)
Evenson et al. (2008)	33 (5–8 years)	≥2296 counts/min	Vertical axis
			(ActiGraph 7164)
Zhu et al. (2013) **	367 (9–17 years)	≥2600 counts/min	Vector-magnitude
			(ActiGraph GT3X)
Hanggi et al. (2013)	49 (10–13 years)	≥3360 counts/min	Vector-magnitude
			(ActiGraph GT3X)
		**steps/min**	
Saunders et al. (2014)	40 (10–17 years)	≥109 steps/min	Triaxial accelerometer
			(Walk4Life MVP)
Harrington et al. (2012)	31 (15.8 ± 0.4 years)	≥100 steps/min	Uniaxial accelerometer
			(ActivPAL)
Tudor-Locke et al. (2018)	22 (6–8 years)	≥128.4 steps/min	Direct Observation
	24 (15–17 years)	≥101.3 steps/min	

* Freedson age- and sex-specific cut-points are derived from (Energy Expenditure (METs) = 5.6832 + 0.00093 (counts/min) − 0.2613 (age in years)) (R^2^ = 0.83). ** Zhu’s cut-points are derived from (Energy Expenditure (kcal/min) = 0.00083 × VM (counts/min) + 0.073 × weight (kg)) based on the weight and RMR (for conversion from energy expenditure to METs) of the current study sample.

**Table 2:** Participant Characteristics (Means ± SD).

Variables	All Children	Sex		Weight Status	
		Boys	Girls	HW	OW
N	41	26	15	30	11
Age (y)	9.6 ± 1.7	9.4 ± 1.9	10.1 ± 1.3	9.7 ± 1.3	9.6 ± 2.7
Weight (kg)	37.9 ± 13.6	35.4 ± 7.5	42.4 ± 19.9	33.5 ± 5.9	50.0 ± 20.5
Height (cm)	141.5 ± 10.1	140.5 ± 9.5	143.1 ± 11.1	140.6 ± 9.0	144.0 ± 12.6
BMI (%ile)	60.0 ± 26.2	60.3 ± 24.6	59.5 ± 29.8	48.2 ± 20.2	92.0 ± 5.2
RMR (kcal/day)	1275 ± 217	1264 ± 203	1295 ± 245	1211 ± 129	1451 ± 307

N, sample size; SD, standard deviation; HW = healthy weight (BMI < 85th percentile); OW = overweight or obese (BMI ≥ 85th percentile); BMI = body mass index; RMR = resting metabolic rate.

**Table 3:** Counts, Cadence, PA Intensity, and PAEE of Children’s Play. Mean (±SEE).

Sample	*n*	Cadence	Accelerometer Counts		PAEE	PA Intensity
		(steps/min)	(V counts/min)	(VM counts/min)	(kcal/min)	(METs)
All	380	66.5 ± 1.3	3335 ± 67	5,350 ± 79	4.8 ± 0.1	6.3 ± 0.1
Girls	143	62.8 ± 2.1 ^a^	2985 ± 115 ^a^	4,831 ± 130 ^a^	4.9 ± 0.1	6.3 ± 0.1
Boys	237	68.7 ± 1.5 ^a^	3546 ± 79 ^a^	5,663 ± 95 ^a^	4.8 ± 0.1	6.3 ± 0.1
HW	276	68.7 ± 1.5 ^b^	3495 ± 75 ^b^	5,546 ± 89 ^b^	4.7 ± 0.1 ^b^	6.4 ± 0.1
OW	104	60.6 ± 2.4 ^b^	2912 ± 135 ^b^	4,830 ± 157 ^b^	5.2 ± 0.2 ^b^	6.1 ± 0.2

^a, b^ = Like symbols are significantly different from each other (*p* < 0.05). SEE, standard error of estimation; *n* = games × children; PA = physical activity; PAEE, physical activity energy expenditure; V = vertical counts/min; VM = vector-magnitude counts/min; MET = metabolic equivalent based on measured resting metabolic rate; HW = healthy weight (BMI < 85th %ile); OW = overweight or obese (BMI ≥ 85th %ile).

**Table 4:** Correlation Coefficients (*r*) among Measured PAEE, PA Intensity, Counts and Cadence.

Measured Values	Accelerometer-Based PA measures		
	V counts/min	VM counts/min	steps/min
PA Intensity (METs)	0.38 *	0.38 *	0.50 *
PAEE (kcal/min)	0.31 *	0.31 *	0.47 *

* *p* < 0.0001. PA = physical activity; V = vertical; VM = vector-magnitude; MET = metabolic equivalent relative to individually measured resting metabolism; PAEE = physical activity energy expenditure.

**Table 5:** Correlation and Kappa Coefficients among Measured vs. Estimated PA Intensity.

Accelerometer-Based Cut-Points	Measured PA Intensity (METs)			
	Pearson *r*	Cohen’s Kappa	Sensitivity	Specificity
Freedson et al. (2005)	0.52	0.49	97.6%	0.8%
Evenson et al. (2008)	0.14	0.05	78.4%	0.8%
Zhu et al. (2013)	0.42	0.37	96.6%	0.8%
Hanggi et al. (2013)	−0.26	−0.02	6.8%	0.3%
Saunders et al. (2014)	0.02	0.0009	4.0%	1.1%
Harrington et al. (2012)	0.04	0.0027	11.3%	1.1%

PA = physical activity; MET = metabolic equivalent relative to individually measured resting metabolism.

## Appendix A

**Table A1:** PAEE, PA Intensity, and Counts and Cadence by Game (Means ± SEE).

Games		Accelerometer Data			PAEE	PA Intensity
	*n*	(V counts/min)	(VM counts/min)	(steps/min)	(kcals/min)	(METs)
Hoop Stations	12	3283 ± 371	5025 ± 351	32.9 ± 4.1	3.0 ± 0.2	4.4 ± 0.2
Monkey in the Middle	13	2126 ± 233	4149 ± 340	33.7 ± 2.9	3.2 ± 0.2	4.6 ± 0.2
Circuit Training	12	2852 ± 405	4855 ± 501	39.6 ± 7.7	3.4 ± 0.3	4.7 ± 0.4
Hot Feet	13	4073 ± 279	6612 ± 382	46.7 ± 3.6	4.0 ± 0.2	5.5 ± 0.2
Pirates’ Treasure	12	2280 ± 379	4266 ± 532	51.6 ± 8.2	4.0 ± 0.4	5.6 ± 0.5
Simon’s Spotlight	15	2535 ± 500	6044 ± 590	56.9 ± 13.4	3.9 ± 0.6	5.9 ± 0.4
Jump the Circuit	13	3960 ± 343	5849 ± 436	48.1 ± 3.6	4.4 ± 0.3	5.9 ± 0.4
Barker’s Hoopla	13	2967 ± 298	4889 ± 380	56.6 ± 5.0	4.6 ± 0.4	5.9 ± 0.4
Scatter Ball	11	4432 ± 346	6212 ± 406	70.7 ± 5.8	4.7 ± 0.3	5.9 ± 0.3
Fox and the Hound	12	2672 ± 326	4628 ± 377	60.2 ± 3.8	4.5 ± 0.3	6.0 ± 0.3
Shooting Stars	11	2579 ± 272	4929 ± 271	80.0 ± 7.2	4.5 ± 0.6	6.0 ± 0.6
Pass the Bacon	14	2923 ± 200	5252 ± 237	60.7 ± 4.5	4.8 ± 0.4	6.1 ± 0.4
Stop and Go	17	3181 ± 236	5288 ± 290	70.2 ± 4.0	4.7 ± 0.4	6.2 ± 0.4
Lumos	13	2544 ± 197	4184 ± 225	76.8 ± 4.3	4.8 ± 0.2	6.2 ± 0.4
Couple Tag	12	3044 ± 192	4737 ± 161	94.4 ± 3.6	5.0 ± 0.3	6.3 ± 0.3
Computer Virus	13	3937 ± 259	5887 ± 216	65.9 ± 2.2	4.9 ± 0.2	6.4 ± 0.3
Slap Ball	11	5135 ± 323	8033 ± 277	52.9 ± 4.1	4.8 ± 0.2	6.5 ± 0.3
Sharks and Minnows	13	2092 ± 223	3944 ± 344	54.5 ± 4.5	4.8 ± 0.4	6.5 ± 0.5
Angels and Devils	11	2841 ± 264	4685 ± 283	73.4 ± 7.1	4.9 ± 0.4	6.5 ± 0.5
Hot Spot	15	3700 ± 255	5373 ± 317	72.6 ± 0.6	5.1 ± 0.4	6.6 ± 0.4
I’m a New Skunk	13	2298 ± 249	4060 ± 348	62.1 ± 5.2	4.8 ± 0.5	6.6 ± 0.6
Soccer	12	3129 ± 340	5348 ± 380	81.9 ± 5.1	5.4 ± 0.5	6.7 ± 0.6
Rat Tail	12	3817 ± 24	5610 ± 263	73.8 ± 2.8	5.4 ± 0.6	6.7 ± 0.3
1-on-1 Showdown	12	3057 ± 228	5442 ± 327	67.3 ± 6.2	5.6 ± 0.5	6.8 ± 0.4
Joker’s Wild	12	4185 ± 339	5657 ± 372	102.2 ± 3.9	5.3 ± 0.4	6.9 ± 0.5
Clean Your Room	14	4911 ± 293	8037 ± 379	45.2 ± 2.0	5.4 ± 0.4	7.1 ± 0.5
Gator Tag	11	3225 ± 183	5098 ± 247	96.2 ± 5.1	5.7 ± 0.3	7.4 ± 0.4
Crazy Soccer	13	3109 ± 224	5246 ± 262	83.6 ± 4.5	5.8 ± 0.4	7.4 ± 0.4
Dragon’s Tail	13	3663 ± 214	5598 ± 226	86.7 ± 4.6	6.0 ± 0.4	7.6 ± 0.4
Me and My Shadow	12	3485 ± 379	5419 ± 387	93.6 ± 8.1	5.6 ± 0.8	7.7 ± 0.7
